# High Levels of Persistent Problem Drinking in Women at High Risk for HIV in Kampala, Uganda: A Prospective Cohort Study

**DOI:** 10.3390/ijerph13020153

**Published:** 2016-01-22

**Authors:** Helen A. Weiss, Judith Vandepitte, Justine N. Bukenya, Yunia Mayanja, Susan Nakubulwa, Anatoli Kamali, Janet Seeley, Heiner Grosskurth

**Affiliations:** 1 MRC Tropical Epidemiology Group, Department of Infectious Disease Epidemiology, London School of Hygiene & Tropical Medicine, London WC1E 7HT, UK; heiner.grosskurth@lshtm.ac.uk; 2 Uganda Research Unit on AIDS, Medical Research Council/Uganda Virus Research Institute (MRC/UVRI), Entebbe, Uganda; jvdpitte@hotmail.co.uk (J.V.); yunia.mayanja@mrcuganda.org (Y.M.); Susan.nakubulwa@mrcuganda.org (S.N.); anatoli.kamali@mrcuganda.org (A.K.); janet.seeley@lshtm.ac.uk (J.S.); 3 Department of Community Health and Behavioral Sciences, School of Public Health, Colleges of Health Sciences, Makerere University, Kampala, Uganda; jbukenya@musph.ac.ug; 4 Department of Global Health and Development, London School of Hygiene & Tropical Medicine, London WC1E 9RE, UK

**Keywords:** alcohol, sex work, Uganda, HIV, problem drinking

## Abstract

The aim of this study was to describe the epidemiology of problem drinking in a cohort of women at high-risk of HIV in Kampala, Uganda. Overall, 1027 women at high risk of HIV infection were followed from 2008 to 2013. The CAGE and AUDIT questionnaires were used to identify problem drinkers in the cohort. Interviewer-administered questionnaires were used to ascertain socio-demographic and behavioural factors. Blood and genital samples were tested for HIV and other sexually transmitted infections. At enrollment, most women (71%) reported using alcohol at least weekly and about a third reported having drunk alcohol daily for at least 2 weeks during the past 3 months. Over half (56%) were problem drinkers by CAGE at enrollment, and this was independently associated with vulnerability (being divorced/separated/widowed, less education, recruiting clients at bars/clubs, and forced sex at first sexual experience). Factors associated with problem drinking during follow-up included younger age, meeting clients in bars/clubs, number of clients, using drugs and HSV-2 infection. HIV prevalence was associated with drinking at enrollment, but not during follow-up. This longitudinal study found high levels of persistent problem drinking. Further research is needed to adapt and implement alcohol-focused interventions in vulnerable key populations in sub-Saharan Africa.

## 1. Introduction

Alcohol use continues to have a significant impact on the HIV epidemic through multiple pathways including increased sexual risk-taking [1], decreased self-care behaviours such as access to HIV services [2], and reduced treatment adherence [3]. As a result of this, alcohol users are estimated to be about twice as likely than the general population to be HIV-infected [4] or to show faster disease progression [5] and alcohol use disorders (AUD) are more common among people living with HIV than among the general population [4,6,7,8].

Uganda is estimated to have one of the highest levels of alcohol consumption *per capita* globally, with an estimated 9.8 L of pure alcohol consumed annually per person aged 15 years or older, compared with 6.0 L for the WHO African Region, and 6.2 L globally [9]. The large proportion (59%) of non-drinkers indicates that people who drink tend to consume substantial amounts of alcohol (23.7 L *per capita* annually in Uganda compared with 16.0 L globally [9]). Prevalence of alcohol use is especially high in vulnerable populations, including women involved in sex work [10] as seen in a recent systematic review which highlighted the need for integrated interventions against alcohol misuse and related adverse effects on physical health, mental health, victimization of violence, and risk of HIV [11].

There are few studies on alcohol use among sex workers and other women at high-risk of HIV infection in sub-Saharan Africa, with only eight of the 52 quantitative studies identified in a systematic review from this region, and none from Uganda. Alcohol use has been consistently identified as a key risk factor for HIV incidence and other sexually transmitted infections (STI) among women at high risk in Eastern Africa [12,13,14,15]. A recent survey of sex workers in Kampala found that alcohol use was associated with higher frequency of physical abuse and forced sex [16]. However, despite the high prevalence of alcohol use and AUD in the region and the strong links with HIV and gender-based violence, interventions to reduce alcohol use have rarely been implemented in sub-Saharan Africa. To our knowledge, there has been only one published randomized controlled trial of a brief alcohol intervention among sex workers in sub-Saharan Africa, which found a significant effect on self-reported alcohol consumption, and sexual violence from clients, after 6 and 12 months of implementation [17].

The aim of this paper is to examine alcohol use disorders in a prospective cohort of women at high-risk of HIV infection in Kampala, Uganda. Specifically, the objectives of the paper are: (i) to compare methods of self-reported drinking behaviours; (ii) to analyse socio-demographic, behavioural and biological factors associated with problem drinking in the cohort at enrollment and during follow-up; and (iii) to assess trends in alcohol use during follow-up.

This is a vulnerable population of women, with high prevalence of HIV at enrollment (37%), high HIV incidence (3.66/100 person-years (pyr)), high prevalence of other sexually transmitted infections (STI) and high prevalence of any alcohol use at enrollment (78%) [15,18]. Previous research showed that problem drinking was strongly associated with HIV incidence (adjusted hazard ratio = 2.85, 95% CI 0.99–8.17, population attributable fraction = 63.5%) in this population [15], and highlighted the key role that alcohol plays in the lives of the participants, with many women meeting clients in bars, and also using the disinhibition effect of alcohol as a strategy to cope with clients, and with the stigma associated with their work [15,19,20].

## 2. Experimental Section 

### 2.1. Study Population

Details of the study population have been published previously [15,18]. Briefly, a closed cohort of 1027 women likely to be involved in high risk sexual behaviour was recruited from Southern Kampala from April 2008–April 2009. The area was mapped to identify “hotspots”, defined as bars, night clubs, local breweries, eating places, lodges and guesthouses known to provide rooms for sex work, or selected street spots often frequented by sex workers in search of clients [18]. Potentially eligible participants were invited to the clinic, in collaboration with a local NGO, Women at Work International (WAWI), who have been offering health education and condom promotion for female sex workers in the area since 2004.

### 2.2. Enrollment and Follow-Up Procedures

Women attending the clinic were screened for eligibility at the dedicated “Good Health for Women” Project (GHWP) clinic in Kampala. All women attending the clinic for screening were offered HIV voluntary counseling and testing, health-education counselling, free condom supplies, and free access to the general care clinic for the total duration of the project, whether or not enrolled into the cohort study. Blood samples testing HIV-positive were sent for confirmation to the MRC clinical laboratories in Entebbe. Women were eligible for enrollment into the cohort if they were aged 18 years or more, self-reported to be involved in commercial sex work (defined as receiving money, goods or other favours in exchange for sex) or employed in entertainment facilities, living or working in Kampala, accepting to undergo the study procedures including collection of blood and genital samples at 3-monthly intervals for testing of HIV and other STI. Women between 15 and 18 years old were eligible if found to be mature minors (*i.e.*, catering for their own livelihood, being pregnant or having already children). Eligible women were scheduled to return for the enrollment visit within one week. Enrolled women were asked to return every 3 months for follow-up visits and whenever they experienced STI symptoms or other health problems.

At each visit, women were interviewed about socio-demographic characteristics, including alcohol use. Questions on alcohol use asked every 3 months were: ever use, drinking alcohol daily for at least 2 weeks during the past 3 months (“in the last 3 months, did you during a period of at least 2 weeks daily drink alcohol?”), binge drinking (“in the last 3 months, how often did you have 6 or more drinks on one occasion?”) and type of alcohol drinks taken (“what type of alcohol do you use most of the time?”). In addition, at enrollment only, the CAGE instrument [21,22] was administered, and at visits from April 2012 onwards, the Alcohol Use Disorders Identification Test (AUDIT) [23] was used. **C**ut Down, **A**nnoyed, **G**uilty, **E**ye-Opener (CAGE) is a screening tool to assess alcohol problems over the lifetime. Problem drinking is defined as a positive response to two of the following questions (“have you ever thought you ought to cut down on drinking?”, “have people annoyed you by criticizing your drinking?”, “have you ever felt bad or guilty about your drinking?”, and “have you ever had a drink first thing in the morning to steady your nerves or to get rid of a hangover?”). The AUDIT is a cross-culturally validated instrument for assessment of alcohol misuse in the general population. The ten-item instrument includes questions to determine patterns of drinking considered harmful, hazardous or symptomatic of dependence in the preceding 12 months, with each item scored as 0–4 points [23]. A score of 8–15 indicates hazardous drinking, 16–19 indicates harmful drinking, and 20–40 indicates alcohol dependence.

At each visit from enrollment, blood was collected and tested for HIV, HSV2 and syphilis. A speculum examination was performed and two endocervical specimens were collected, one for the diagnosis of gonococcal and chlamydial infection and one for diagnosis of *Mycoplasma genitalium* infection. One high vaginal specimen was collected and inoculated for culture of *Trichomonas vaginalis*, another to prepare a slide for the detection of bacterial vaginosis and of *Candida* ssp. infection. From April 2011 onwards, due to financial constraints, molecular amplification assays and culture tests were no longer performed routinely for any of the STIs. Other procedures remained unchanged. Women with symptomatic STIs were treated syndromically immediately. Women with asymptomatic STIs were treated within days after laboratory results became available. Education on HIV/STI, risk reduction counselling including demonstration and promotion of safe condom use were provided face-to-face at every visit and in group sessions throughout follow-up.

### 2.3. Alcohol Counselling

Specific counselling on alcohol drinking was introduced in April 2012, to address the wide-spread problem of alcohol use disorders in the cohort. The single-session intervention was delivered at the clinic by project staff, after administration of the AUDIT, and included:

(i) General education about harmful consequences of excessive alcohol use e.g., liver disease and cancer, psychiatric disorders, increased risk of HIV infection, reduced life expectancy, and alcohol dependence;
(ii) Specific messages depending on AUDIT score results:
  - Non-drinkers (score 0) were encouraged to continue abstaining from alcohol and such sessions were usually very short (around 5 min).
  - Non-hazardous drinkers (score 1–7) were encouraged to keep alcohol consumption at a minimum, with emphasis on the potential social, legal and chronic health problems due to drinking. These sessions took 10–20 min.
  - Hazardous, harmful and dependent drinkers (score ≥ 8) were encouraged to take immediate action to reduce the risk associated with their drinking. They were supported to acknowledge their addiction and its possible consequences. The counselling session covered a range of physical and psycho-social issues including health and nutrition, family, spiritual needs and employment, and the possible benefits of reducing their alcohol intake. These sessions took an average of 45 min.

### 2.4. Laboratory Procedures

Serum specimens were tested for antibodies against HIV-1 (Abbott Determine HIV-1/2 with confirmation by two independent ELISA tests: Vironostika Uniform II plus O, Murex HIV 1.2.O), HSV-2 (IgG ELISA test, Kalon Biological Ltd., Guildford, UK) and for syphilis (RPR Biotec and TPHA Biotec, Kentford, UK). *Neisseria gonorrhoeae* (NG) and *Chlamydia trachomatis* (CT) were diagnosed on endocervical specimens using the Amplicor PCR test (Roche Diagnostic Systems Inc., Branchburg, NJ, USA) and *Trichomonas vaginalis* (TV) was detected using a commercial culture kit (InPouch TV, BioMed Diagnostics, White City, OR, USA). Microscopy on a gram-stained vaginal specimen was performed to diagnose bacterial vaginosis (using Nugent’s criteria) and candidiasis. Laboratory testing for these infections was performed at the central laboratories of the MRC/UVRI Uganda Research Unit on AIDS in Entebbe. For the diagnosis of *Mycoplasma genitalium*, endocervical specimens, collected using Cobas Amplicor STM collection tubes (Roche Diagnostic Systems Inc.), were tested using a commercially available Real-TM PCR assay (Sacace Biotechnologies, Como, Italy) at the Centre for HIV and Sexually Transmitted Infections, National Institute for Communicable Diseases, National Health Laboratory Service in Johannesburg. Further details of this PCR test have been reported previously [18].

### 2.5. Statistical Analysis

Data were double-entered in Access and analyzed using STATA 13.0 (Stata Inc., College Station, TX, USA). Logistic regression was used to estimate prevalence ratios (PRs; for enrollment analyses) or risk ratios (RRs; for follow-up analyses) associated with problem drinking using the marginal standardization technique with 95% confidence intervals (CI) estimated via the delta method [24]. Socio-demographic and behavioural factors associated with problem drinking (defined by CAGE) at enrollment (at a significance level *p* < 0.10) were included in a multivariable logistic regression model and a core group of factors independently associated (*p* < 0.10 after adjustment) with problem drinking were retained in the final model. The association of each reproductive tract infection and STI with problem drinking was adjusted for the core group of socio-demographic and behavioural factors.

Chi-squared statistics were used to compare proportions of self-reported problem drinkers by CAGE with other reported drinking behaviour at enrollment. Questions on daily alcohol use and binge drinking were asked at every visit, and random effects logistic regression analyses were used to estimate prevalence ratios for these outcomes, adjusting for within-woman correlation. The models to identify risk factors for these outcomes were built up as for the baseline associations with problem drinking, by first creating a core group of socio-economic and behavioural factors independently associated with the alcohol variables (*p* < 0.10 after adjustment) and adjusting all variables for this core group. Time-updated variables were used in this analysis. Trends in alcohol use patterns over time (drinking in the past 3 months, daily alcohol use and binge drinking, AUDIT score ≥ 8) were analyzed using random effects logistic regression (binary outcomes) or mixed effect linear regression (AUDIT score), adjusting for within-person clustering, with months-since-enrollment as an independent variable.

### 2.6. Ethical Considerations

Informed consent was obtained from all participants. The study was approved by the Research Ethics Committee of the Uganda Virus Research Institute, the Uganda National Council for Science and Technology (HS 364) and the Ethics Committee of the London School of Hygiene and Tropical Medicine (5198). Throughout the study, free primary health care was offered to participating women and their children aged <5 years old. All participants with HIV infection had their CD4-count measured, and women eligible for antiretroviral therapy (ART) were referred to an accredited HIV-care centre until January 2013, and thereafter treated at the clinic. Those not eligible were provided with Cotrimoxazole prophylaxis and their CD4-count was routinely monitored.

## 3. Results and Discussion

### 3.1. Characteristics of the Cohort

The mean age of the 1027 participants at enrollment was 26 years (standard deviation (sd) ±5.7 years). Levels of education were low: 49% of the women had never been to school or had not completed primary school and only 10% attended secondary school. The majority (70%) were divorced, separated or widowed, and 91% financially supported dependents. The place of recruiting clients varied from entertainment facilities such as bars, clubs or discos (39%), the street (16%), or several of these places (38%). Sex work was the sole source of income for 34% of the participants, 62% had income besides sex work and 5% were employed in an entertainment facility but reported not being involved in sex work.

In the current paper, we analyse data from enrollment (April 2008–April 2009) to 30 September 2013. The median follow-up time was 4.3 years (range 0–5.5 years). Retention in the cohort was relatively high for the first 3 years of follow-up (92% of women attending at 1 year, 85% at 2 years, 79% at 3 years) but then reduced, with 63% at 4 years post-enrollment and 45% seen at or after 30 September 2013.

HIV prevalence was 37% at enrollment, and HIV incidence was 2.77/100 (pyr) over the total follow-up period (95% CI 2.14–3.60/100 pyr) but decreased substantially with time-in-study, from 6.80/100 pyr in the period April 2008–March 2009 to 1.17/100 pyr in the period April 2012–September 2013 (age-adjusted *p*-value for trend < 0.001).

### 3.2. Comparison of Self-Reported Alcohol Use at Enrollment

At enrollment, the majority of participants reported using alcohol (*n* = 803, 78%), with 71% reporting alcohol use at least once a week (Table 1). Among the 803 drinkers, 28% used alcohol both during work and outside work, with 38% drinking mainly out of work and 34% drinking mainly during work. Almost all women (94%) drank beer, either bottled beer only (54%), or as one of several types of alcohol (bottled beer, local beer and spirits; 40%).

Problem drinking was commonly reported, with a third of all women (32%) reporting having drunk alcohol daily for a period of at least 2 weeks during the past 3 months, a quarter (26%) categorized as binge drinkers (6 or more drinks on the same occasion during the past 3 months), and a similar proportion (24%) reporting not being able to stop drinking once they had started (Table 1). Over half of the women responded “yes” to the question “Have you ever considered yourself to have a drinking problem or to be an excessive drinker?” (53%), and a similar proportion were classified as problem drinkers on the CAGE questionnaire (56%; Table 1).

Table 1 compares specific self-reported drinking behaviours with problem drinking defined by CAGE. Participants classified as problem drinkers by CAGE were more likely than non-problem drinkers to report each risky drinking behavior: using alcohol daily for a 2 week period in the past 3 months (47% *vs.* 26%), have 6 or more drinks on one occasion (40% *vs.* 17%), be unable to stop drinking once they had started (38% *vs.* 12%) or to consider themselves to have a drinking problem (83% *vs.* 40%). Forty percent of women classified as non-problem drinkers on CAGE self-reported themselves to have a drinking problem.

**Table 1:** Self-reported drinking patterns at enrollment, by problem drinking (defined by CAGE).

Reported Drinking Behaviour	All Women (Including Non-Drinkers)	Alcohol Users *N* = 803	Non Problem Drinkers (by CAGE) *N* = 231	Problem Drinkers (by CAGE) *N* = 572	*p*-Value (Problem Drinkers *vs.* Non Problem Drinkers)
	*n* (%)	*n* (%)	*n* (%)	*n* (%)	
Drinks alcohol	803 (78%)	803 (100%)	231 (100%)	572 (100%)	n/a
Drinks at least once a week	732 (71%)	732 (91%)	189 (42%)	543 (95%)	*p* < 0.0001
Daily alcohol use for a period of at least 2 weeks during the past 3 months	331 (32%)	331 (41%)	60 (26%)	271 (47%)	*p* < 0.0001
6 or more drinks on one occasion in the last three months	269 (26%)	269 (34%)	39 (17%)	230 (40%)	*p* < 0.0001
Unable to stop drinking once started	247 (24%)	247 (31%)	28 (12%)	219 (38%)	*p* < 0.0001
Considered themselves to have a drinking problem or to be an excessive drinker	546 (53%)	546 (68%)	92 (40%)	454 (83%)	*p* < 0.0001
Problem drinkers defined by CAGE	572 (56%)	572 (71%)	n/a **^a^**	n/a **^b^**	n/a

**^a^** Zero by definition; **^b^** *n* = 572 by definition.

### 3.3. Factors Associated with Problem Drinking at Enrollment

Table 2 shows factors associated with problem drinking defined by CAGE at enrollment. Compared with non-drinkers and non-problem drinkers, women with problem drinking defined by CAGE tended to be older, have previously been married, have low levels of education, provide financial support to dependents, currently practice sex work, have a larger number of lifetime partners, their first sexual experience being forced sex, to have had more partners in the past month, had a paid client in the past month, greater number of clients in the past month and practice inconsistent condom use (Table 2). On multivariable analysis, independent factors associated with problem drinking included being divorced, separated or widowed compared with being single (adjusted PR (aPR) = 1.18, 95% CI 1.02–1.38), having less than complete primary education (aPR = 1.16, 95% CI 1.04–1.30), recruiting clients from bars, clubs or several sites, and reporting rape as the first sex act (aPR = 1.26, 95% CI 1.03–1.54 compared with a husband/fiancé; Table 2).

**Table 2:** Socio-demographic and behavioural factors associated with alcohol use disorders at enrollment.

Factor	*N* (%)	% with Problem Drinking	Prevalence Ratio	95% CI	Adjusted Prevalence Ratio ^a^	95% CI
**Socio-economic factors**				**		**
**Age Group**				*p* = 0.1		*p* = 0.2
14–24 Years	412 (40%)	53%	1		1	
25–34 Years	505 (49%)	59%	1.12	0.99–1.25	1.02	0.91–1.15
35+ Years	110 (11%)	51%	0.96	0.78–1.18	0.86	0.69–1.07
**Marital Status**				*p* = 0.0008		*p* = 0.004
Single	229 (22%)	50%	1		1	
Married	83 (8%)	41%	0.82	0.62–1.10	0.85	0.63–1.14
Divorced, separated or widowed	715 (70%)	59%	1.19	1.03–1.38	1.18	1.02–1.38
**Religion**				*p* = 0.02		*p* = 0.04
Catholic	440 (43%)	55%	1		1	
Anglican	284 (28%)	60%	1.09	0.96–1.24	1.12	0.99–1.27
Muslim	265 (26%)	55%	0.99	0.86–1.14	0.99	0.86–1.14
Other	38 (4%)	34%	0.62	0.40–0.97	0.69	0.45–1.06
**Ethnicity**				*p* = 0.4		*p* = 0.3
Muganda	608 (60%)	55%	1		1	
Other Ugandan	371 (36%)	58%	1.06	0.95–1.19	1.03	0.92–1.16
Not Ugandan	46 (4%)	50%	0.92	0.68–1.23	0.82	0.59–1.14
**Education level**	****			*p* = 0.006		*p* = 0.008
At least primary completed	524 (51%)	52%	1		1	
Less than primary completed	503 (49%)	60%	1.17	1.04–1.30	1.16	1.04–1.30
**Provides financial support to others**				*p* = 0.05		*p* = 0.2
No	95 (9%)	46%	1		1	
Yes	932 (91%)	57%	1.22	0.98–1.53	1.15	0.92–1.44
**Has a regular partner**	****			*p* = 0.4		0.9
Yes	752 (27%)	55%	1		1	
No	275 (73%)	58%	1.05	0.93–1.19	0.99	0.87–1.12
**Source of income**	****			*p* = 0.1		*p* = 0.9
Sex work alone	346 (34%)	56%	1		1	
Sex work and other job	633 (62%)	57%	1.01	0.90–1.13	1.01	0.89–1.15
No sex work	48 (5%)	42%	0.74	0.52–1.05	-	-
**Cost per sex act (Schillings)**	****			*p* = 0.2		*p* = 0.4
<5000	231 (24%)	60%	1		1	
≥5000	748 (76%)	55%	0.92	0.81–1.04	0.94	0.83–1.07
**Place of recruiting clients**	****			*p* = 0.002		*p* = 0.007
Bar, club or restaurant	383 (39%)	58%	1		1	
Street	152 (16%)	50%	0.87	0.72–1.04	0.85	0.71–1.01
Other	70 (7%)	39%	0.67	0.49–0.91	0.70	0.52–0.94
Several	374 (38%)	61%	1.06	0.94–1.19	1.02	0.91–1.15
**Behavioural factors**	****			**		**
**Number of lifetime partners**	****			*p* = 0.002		*p* = 0.2
<20	129 (13%)	42%	1		1	
20–49	155 (17%)	61%	1.46	1.15–1.86	1.25	0.98–1.60
50 or can’t remember	743 (72%)	57%	1.36	1.10–1.68	1.16	0.93–1.45
**Age at first sex (years)**				*p* = 0.28		*p* = 0.55
≤14	355 (36%)	58%	1		1	
15–22	637 (64%)	55%	0.94	0.84–1.05	0.96	0.85–1.09
**First sexual partner**	****			*p* = 0.1		*p* = 0.08
Husband/fiancé	257 (25%)	54%	1		1	
Boyfriend/lover	657 (64%)	56%	1.03	0.91–1.18	1.12	0.97–1.29
Casual Aquaintance	31 (3%)	45%	0.84	0.56–1.26	0.87	0.58–1.31
Forced sex	82 (8%)	67%	1.25	1.03–1.51	1.26	1.03–1.54
**Behavioural factors**	****			**		**
**Number of partners in last month**	****	****		*p* = 0.08		*p* = 0.6
0–4	323 (31%)	50%	1		1	
5–19	307 (30%)	57%	1.13	0.98–1.31	1.01	0.87–1.18
20–49	236 (23%)	61%	1.21	1.04–1.40	1.11	0.94–1.30
50+ or can’t remember	161 (16%)	58%	1.15	0.97–1.37	1.04	0.86–1.26
**Paying client in last month ^b^**				*p* = 0.03		*p* = 0.8
No	122 (12%)	47%	1		1	
Yes	905 (88%)	59%	1.22	1.00–1.48	1.03	0.83–1.28
**Number of clients in last month**	****			*p* = 0.07		*p* = 0.8
<5	353 (34%)	51%	1		1	
5–49	512 (50%)	58%	1.15	1.01–1.30	1.04	0.91–1.20
>50 or can’t remember	162 (16%)	59%	1.16	0.98–1.36	1.05	0.88–1.26
**Inconsistent condom use**	****			*p* = 0.08		*p* = 0.2
No	541 (60%)	55%	1		1	
Yes	364 (40%)	60%	1.11	0.99–1.24	1.09	0.97–1.22

**^a^** Adjusted for marital status, religion, education level, place of recruiting clients and type of first partner; **^b^** Excluding the 48 women who reported not to be sex workers.

Table 3 shows the association of HIV/STI infection with problem drinking at enrollment, compared with a combined group of non-drinkers and non-problem drinkers, adjusted for socio-demographic factors. There was some evidence of an association of problem drinking with HIV infection (aPR = 1.12, 95% CI 0.95–1.33), HSV-2 (aPR = 1.06, 95% CI 0.99–1.13), *Candida albicans* (aPR = 1.42, 95% CI 0.97–2.06), *Neisseria gonorrhoeae* (aPR = 1.39, 95% CI 0.99–1.95) and *Mycoplasma genitalium* infections (aPR = 1.41, 95% CI 1.02–1.06).

Further analyses of the association with HIV infection at enrollment showed that both non-problem drinkers and problem drinkers were at increased risk of HIV compared with non-drinkers (aPR = 1.39, 95% CI 1.05–1.95; aPR = 1.36, 95% CI 1.06–1.74 respectively) but there was no evidence that problem drinkers were at increased risk compared with non problem-drinkers (excluding non-drinkers) (39.8% *vs.* 39.4%; aPR = 0.98, 95% CI 0.80–1.19).

**Table 3:** Association of problem drinking by CAGE criteria with reproductive tract infections and sexually transmitted infections, at enrollment.

Factor	*n*/*N*	Percent with Outcome	Adjusted Prevalence Ratio ^a^	95% CI
**HIV-positive**				*p* = 0.2
Non problem-drinkers **^b^**	156/455	34%	1	
Problem drinkers	225/572	39%	1.13	0.95–1.35
**HSV-2**				*p* = 0.07
Non problem-drinkers	348/455	76%	1	
Problem drinkers	474/572	83%	1.06	0.99–1.13
**Active syphilis ^c^**				*p* = 0.4
Non problem-drinkers	42/455	9%	1	
Problem drinkers	61/569	11%	1.19	0.81–1.75
**Bacterial vaginosis ^d^**				*p* = 0.5
Non problem-drinkers	246/455	54%	1	
Problem drinkers	327/572	57%	1.04	0.92–1.17
***Candida* ssp.**				*p* = 0.07
Non problem-drinkers	41/455	9%	1	
Problem drinkers	71/572	12%	1.42	0.97–2.06
***Trichomonas vaginalis***				*p* = 0.4
Non problem-drinkers	81/455	18%	1	
Problem drinkers	95/572	17%	0.90	0.68–1.18
***Neisseria gonorrhoeae*^e^**				*p* = 0.06
Non problem-drinkers	49/454	11%	1	
Problem drinkers	85/572	15%	1.39	0.99–1.95
***Chlamydia trachomatis*^f^**				*p* = 0.6
Non problem-drinkers	41/454	9.0%	1	
Problem drinkers	51/572	8.9%	1.12	0.74–1.69
***Mycoplasma genitalium*^g^**				*p* = 0.04
Non problem drinkers	54/453	12%	1	
Problem drinkers	94/572	16%	1.41	1.02–1.96

**^a^** Adjusted for marital status, religion, education level, place of recruiting clients and type of first partner; **^b^** The baseline group includes both non-drinkers and drinkers who are not problem drinkers; **^c^** Defined as having TPHA-positive and RPR-positive serological results. Missing data for three participants; **^d^** Outcome is BV positive vs intermediate/negative bacterial flora; **^e^** Missing data for one participant; **^f^** Missing data for one participant; **^g^** Missing data for two participants.

### 3.4. Trends in Self-Reported Alcohol Use During Follow-Up

The proportion of women who said they had used alcohol in the past 3 months decreased from 78% at enrollment to 65% at Year 1, 62% at Year 2, 60% at Year 3, and 58% at Year 4 (*p*-trend adjusted for within-person clustering <0.0001). The single-session counselling intervention was introduced in April 2012 (Year 3–4), by which time prevalence had already decreased, but there was little evidence of a further decrease after this time. The proportion of women who drank daily decreased slightly during follow-up from 32% at enrollment to 24% at month 3, 21% at Year 2, and 15% at Year 4 (*p*-trend < 0.0001). Similarly, the proportion of binge drinkers declined slightly from 26% at enrollment to 12%–15% at Years 2–4 (*p*-trend < 0.0001). Given the loss to follow-up, there is potential for bias in these estimates. Participants who were followed for less than the median time (4.3 years) were more likely to be single, not financially support others, recruit in several places and be HIV negative at enrollment (results not shown).

The AUDIT was administered to 721 (70%) of participants during follow-up (from February 2012). Using the first AUDIT score administered, 27% of participants were classified as hazardous drinkers (AUD 8–15), 8% as harmful drinkers (AUD 16–19), and 11% as dependent drinkers (AUD ≥ 20). Comparison with CAGE showed that, of those classified as problem drinkers by the CAGE test at enrollment, 56% were hazardous, harmful or dependent drinkers during follow-up on the AUDIT, compared to 35% of non-problem drinkers at enrollment (*p* < 0.001). Similar to the other self-reported drinking behaviours, the mean AUDIT score decreased slightly from 7.2 at month 36 when the test was first applied, to 6.3 at month 60 (*p*-trend < 0.001), as did the proportion of participants with an AUDIT score of 8 or above (*p*-trend = 0.002).

### 3.5. Factors Associated with Daily Drinking and Binge Drinking During Follow-Up

Factors associated with daily drinking (drinking alcohol daily for at least 2 weeks during the past 3 months) and binge drinking respectively, during follow-up are shown in Table 4. As described in Section 3.4, both daily drinking and binge drinking decreased significantly during the study. Other factors independently associated with these drinking behaviours were being a sex worker, meeting clients in a bar or club, having a large number of lifetime clients and recent clients, and using drugs. In addition, binge drinking was associated with younger age, religion (being non-Catholic/Anglican) and HSV-2 infection at the previous visit.

There was some evidence that HIV positive individuals reduced their drinking behavior more than HIV negative individuals - being HIV positive at the previous visit was associated with a slightly reduced risk of daily or binge drinking (daily drinking: aPR = 0.81, 95% CI 0.62–1.08; binge drinking: aPR = 0.78, 95% CI 0.60–1.03; Table 4) compared with those who were HIV negative, and the decreasing trend in risky drinking with time was significantly stronger among HIV positive than HIV negative individuals (*p* < 0.001).

### 3.6. Discussion

This study shows high prevalence of persistent problem drinking in this cohort of women at high risk for HIV-infection in Kampala, with over half of participants scoring as problem drinkers by the CAGE test at enrollment and 47% scoring as hazardous, harmful or dependent drinkers during follow-up by the AUDIT. After an initial decline in prevalence, a relatively high prevalence in the cohort persisted despite regular contact of participants with health educators and administration of a single-session alcohol-focused counselling intervention, and this was closely linked with meeting clients in bars and having a larger number of clients.

The initial decline seen in the current study may be due reductions in drinking among both HIV negative and HIV positive women, due to contact with clinic staff. These results support a study among participants attending HIV counselling and testing (HCT) at Mulago Hospital, Kampala, where self-reported alcohol consumption declined in the first 3 months following HCT, among both those testing HIV positive and HIV negative [25]. Other studies have discussed “assessment reactivity” in response to the CAGE or AUDIT, whereby assessment of alcohol consumption itself increases the self-awareness of alcohol problems, and thus triggers a reduction in alcohol consumption [26]. The effect of this in sex worker populations may plausibly be less than in general populations due to the socio-economic and other reasons driving their alcohol use [19].

**Table 4:** Association of daily drinking and binge drinking during follow-up with socio-economic, behavioural and biological factors.

Factor	% of Visits with Daily Drinking Reported	Adjusted Risk Ratio ^a^ (95% CI)	% of Visits with Binge Drinking Reported	Adjusted Risk Ratio ^b^ (95% CI)
**Calendar period**		*p* < 0.0001		*p* < 0.0001
April 2008–September 2008	28%	1	24%	1
October 2008–March 2009	27%	0.87 (0.64–1.19)	22%	0.92 (0.68–1.25)
April 2009–September 2009	23%	0.65 (0.51–0.89)	15%	0.42 (0.31–0.58)
October 2009–March 2010	22%	0.59 (0.43–0.80)	13%	0.33 (0.24–0.46)
April 2010–September 2010	18%	0.42 (0.30–0.58)	10%	0.26 (0.19–0.36)
October 2010–March 2011	20%	0.57 (0.41–0.79)	12%	0.36 (0.26–0.51)
April 2011–September 2011	19%	0.52 (0.38–0.74)	14%	0.50 (0.37–0.70)
October 2011–March 2012	19%	0.60 (0.43–0.84)	13%	0.46 (0.33–0.64)
April 2012–September 2012	16%	0.43 (0.29–0.64)	16%	0.77 (0.54–1.12)
**Socio-economic factors**		**		**
**Age Group**		*p* = 0.7		*p* = 0.01
14–24 years	20%	1	17%	1
25–34 years	23%	1.19 (0.84–1.68)	15%	0.72 (0.53–1.00)
35+ years	19%	1.12 (0.64–1.94)	8%	0.42 (0.24–0.73)
**Religion**		*p* = 0.1		*p* = 0.002
Catholic	22%	1	15%	1
Anglican	21%	0.79 (0.54–1.16)	17%	1.23 (0.85–1.79)
Muslim	21%	0.64 (0.43–0.95)	14%	0.71 (0.47–1.05)
Other	15%	0.49 (0.19–1.24)	6%	0.19 (0.08–0.51)
**Source of income**		*p* < 0.001		*p* < 0.001
Sex work alone	26%	1	18%	1
Sex work and other job	24%	1.12 (0.94–1.32)	17%	1.21 (1.01–1.48)
No sex work	7%	0.25 (0.19–0.33)	5%	0.40 (0.26–0.56)
**Behavioural factors**		**		**
**Place of recruiting clients**		*p* < 0.001		*p* < 0.001
Bar, club or restaurant	28%	1	19%	1
Street	20%	0.75 (0.59–0.96)	13%	0.79 (0.59–1.06)
Other	12%	0.56 (0.45–0.70)	9%	0.58 (0.45–0.75)
Several	32%	1.02 (0.88–1.19)	23%	1.10 (0.92–1.31)
**Number of lifetime partners**		*p* < 0.001		*p* = 0.1
<20	11%	1	10%	1
20–49	14%	0.90 (0.46–1.75)	11%	0.87 (0.46–1.65)
≥50 or can’t remember	25%	1.83 (1.07–3.13)	16%	1.41 (0.83–2.41)
**Number of partners in the last month**		*p* < 0.001		*p* < 0.001
0–4	13%	1	9%	1
5–19	27%	1.82 (1.51–2.20)	18%	1.63 (1.32–2.03)
20–49	35%	2.72 (2.19–3.38)	23%	2.78 (2.16–3.58)
≥50 or can’t remember	38%	3.05 (2.35–3.96)	28%	3.02 (2.23–4.11)
**Biological factors**		**		**
**Used drugs of addiction**		*p* < 0.001		*p* < 0.001
No	17%	1	12%	1
Yes	39%	1.93 (1.64–2.28)	26%	1.75 (1.46–2.09)
**HIV at previous visit**		*p* = 0.2		*p* = 0.6
No	24%	1	17%	1
Yes	18%	0.81 (0.62–1.08)	12%	0.78 (0.60–1.03)
**HSV2 at previous visit**		*p* = 0.2		*p* = 0.002
No	19%	1	13%	1
Yes	22%	1.26 (0.87–1.81)	15%	1.76 (1.22–2.56)

**^a^** Adjusted for time period, source of income, place of recruitment, number of lifetime partners and number of partners in the last month; **^b^** Adjusted for time period, age, religion, source of income, place of recruitment and number of partners in the last month.

Our study adds to the few longitudinal studies of alcohol use among sex workers in Africa [13,27] and is the first cohort study we are aware of to include both HIV-positive and HIV-negative women. The level of problem drinking in our study is higher than seen in studies of women employed in food and recreational facilities in Tanzania (prevalence of 20%–37% by CAGE) [14,27,28,29,30,31,32,33,34,35,36,37]. This may reflect the higher proportion of women in the Kampala cohort who were involved with transactional sex, and higher levels of alcohol use in Uganda compared with many other African countries [9]. It also shows the high prevalence of alcohol use in this group compared with the general female population in Uganda, in which an estimated 0.3% of women aged 15 years and above engaged in heavy episodic drinking and only 0.7% are alcohol dependent [9].

Strengths of our study include the large size of the cohort, high retention in the first 3 years, regular and frequent follow-up, the combination of socio-demographic, behavioural, and biological data and the use of internationally recommended screening tests to assess AUD at the individual level. Another strength is the longitudinal design which enables the observation of temporal relationships. Our previous work showed that alcohol use was a strong risk factor for increased HIV incidence [15], but in the current analysis, HIV infection at the previous visit was associated with a slightly reduced risk of daily or binge drinking. We found evidence of a greater decline in drinking among participants living with HIV than HIV negative participants. This contrasts with the data on HSV-2 infection at the previous visit, which was associated with an increased risk of both daily drinking and binge drinking. The results suggest that women who are living with HIV may modify their risky drinking behaviour, especially once on ART, or may be partly due to reporting bias if the women think it is better not to report heavy drinking whilst on ART. This supports findings from a recent cohort study of HIV-infected adults in Mbarara, Uganda, where reported alcohol consumption declined in the 3 months following ART initiation [38]. A limitation of our study was that we did not have data on HIV treatment and care. However, the cohort is now being extended, and HIV treatment is provided on site, so further research into this area will be possible. Further research into the dynamics of alcohol use and HIV infection, treatment and care, including qualitative work to address reporting biases such as social desirability bias would be useful.

Another limitation of the study was that the AUDIT was not applied at enrollment or during the first 3 years of follow up, and that no other “gold standard” measure of alcohol use, such as the DSM-IV Scheduled Clinical Interview or a biological marker was used. It is notable that a substantial minority (40%) of participants who did not score as problem drinkers on the CAGE questionnaire self-identified as a problem drinker, indicating that the CAGE may not be identifying all problem drinkers in this population and that further tools may need to be developed to better capture drinking problems in this setting. Three recent studies have evaluated self-reported alcohol use against biomarkers in African populations, with mixed results [39,40,41] and further studies evaluating self-reported alcohol measures (including AUDIT and CAGE, but also more detailed methods such as Time Line Follow-Back) against alcohol biomarkers such as phosphatidylethanol (PEth) are needed.

The decline in reported problem drinking over time may have partly been subject to misclassification due to social desirability bias of the interviewer-administered questionnaire that may have affected participants’ responses. It may also have been assessment reactivity, *i.e.*, that administration of the AUDIT may have led to reductions in alcohol consumption [42,43], or due to biases associated with loss to follow-up. A further limitation of the study is that no complete sampling frame was available as the population is hard-to-reach and enumerate, and this may limit the generalizability of the findings. Further, this study was a closed cohort with four years of follow-up and participants had been exposed to educational and STI interventions for this period which may make the results less generalizable to women at high-risk in Kampala; and although retention was fairly high overall, loss-to follow up had reached 37% by year 4. We conducted a complete case analysis, and analysis by duration of follow-up showed that place of recruitment was associated with both shorter duration in follow-up and risky drinking behavior, so we may be under-estimating the persistence of drinking in the cohort.

In addition to posing a public health problem itself, alcohol is associated with an increased risk of HIV infection [4,15] and with poorer adherence to ART [3,44,45]. Improved understanding of alcohol use patterns in high-risk populations is essential to design effective interventions to improve adherence to ART. Our previous qualitative research has shown that, in this cohort, alcohol use is driven by the emotional and economic needs of the participants, and also promoted by clients who encourage consumption [19,20]. Many sex workers only started drinking alcohol when they joined sex work on the advice of more experienced peers as a way to cope with the job. Almost all the women who took alcohol regularly admitted that they had a higher chance of engaging in unsafe sex when they drank, blaming alcohol use for unsafe sex, acts of violence and poor decision making which increased sexual and physical violence. These qualitative findings are consistent with our quantitative findings in this paper, that prevalence of problem drinking is highest among the most vulnerable women in terms of socio-economic status (divorced/widowed, little education), those who worked in bars, and those whose first experience of intercourse had been forced sex. Similar findings were seen in Tanzania [28,29] and Kenya [13], and fit with alcohol playing a role in either causing marital breakdown, or resulting from it. Problem drinking was also associated with an increased prevalence of HIV, HSV-2, *Candida* ssp., *Neisseria gonorrhoeae* and *Mycoplasma genitalium* infections at enrollment, emphasizing the link between alcohol use and risky sexual behaviour.

These results highlight the urgent need for specific alcohol-focused interventions in this vulnerable population, in line with a recent WHO report on alcohol and global health which calls for further development and implementation of measures to reduce alcohol-related harm, to be guided by public health interests and the best available evidence [9]. A review of policy-relevant strategies and interventions to address the burden of alcohol on individuals and society in South Africa considered that the strategies most likely to work were improving the enforcement of existing legislation/regulations regarding the minimal age of purchase, drinking and driving, and implementing additional structural interventions such as increasing total tax on alcohol products and implementing restrictions on alcohol marketing [46]. In addition to structural interventions, which are challenging to implement, community- and individual-level interventions are needed. A cornerstone of AUD management are brief interventions which comprise a group of psychological treatments [47], including those based on motivational interviewing (MI). A meta-analysis in 2003 found that adaptations of MI showed clinical impact on alcohol use [48], but none of these studies had been conducted in sub-Saharan Africa. A more recent systematic review of adherence-enhancing interventions for ART in sub-Saharan Africa identified MI for alcohol-dependent patients as the only one of 21 identified strategies to find a significant impact on adherence [49]. A recent study in South Africa also showed that it was feasible to implement a combined alcohol and HIV prevention intervention in bar settings, consisting of peer-led brief interventions based on MI, delivered to bar patrons [50]. Another recent systematic review of interventions to reduce HIV transmission in the context of sex work also highlighted the need to develop and evaluate effective alcohol-control strategies [51], and notably, of the 26 intervention studies identified among female sex workers in sub-Saharan Africa only one was aimed at the adoption of safer drinking patterns [52]. This counselling and education intervention found a decrease in substance use during sex work and of reported STI symptoms in the intervention group [52].

## 4. Conclusions

This study provides insight into longitudinal alcohol consumption patterns among women at high risk of HIV infection, and the high prevalence of both persistent problem drinking and HIV infection found in this cohort of women in Kampala adds urgency to the need to develop and evaluate interventions in the context of sex work in Africa, through trials incorporating individual, community and structural level interventions.

## References

[1] Rehm J., Shield K.D., Joharchi N., Shuper P.A. (2012). Alcohol consumption and the intention to engage in unprotected sex: Systematic review and meta-analysis of experimental studies. Addiction.

[2] Gari S., Doig-Acuna C., Smail T., Malungo J.R., Martin-Hilber A., Merten S. (2013). Access to HIV/AIDS care: A systematic review of socio-cultural determinants in low and high income countries. BMC Health Serv. Res..

[3] Mayston R., Kinyanda E., Chishinga N., Prince M., Patel V. (2012). Mental disorder and the outcome of HIV/AIDS in low-income and middle-income countries: A systematic review. Aids.

[4] Fisher J.C., Bang H., Kapiga S.H. (2007). The association between HIV infection and alcohol use: A systematic review and meta-analysis of african studies. Sex. Transm. Dis..

[5] Neuman M.G., Schneider M., Nanau R.M., Parry C. (2012). Alcohol consumption, progression of disease and other comorbidities, and responses to antiretroviral medication in people living with HIV. AIDS Res. Treat..

[6] Kalichman S.C., Simbayi L.C., Kaufman M., Cain D., Jooste S. (2007). Alcohol use and sexual risks for HIV/AIDS in sub-Saharan Africa: Systematic review of empirical findings. Prev. Sci..

[7] Baliunas D., Rehm J., Irving H., Shuper P. (2010). Alcohol consumption and risk of incident human immunodeficiency virus infection: A meta-analysis. Int. J. Public Health.

[8] Pithey A., Parry C. (2009). Descriptive systematic review of sub-Saharan African studies on the association between alcohol use and HIV infection. SAHARA J..

[9] World Health Organization Global Status Report on Alcohol and Health 2014. http://apps.who.int/iris/bitstream/10665/112736/1/9789240692763_eng.pdf.

[10] Plant M.L., Plant M.A., Peck D.F., Setters J. (1989). The sex industry, alcohol and illicit drugs: Implications for the spread of HIV infection. Brit. J. Addict..

[11] Li Q., Li X., Stanton B. (2010). Alcohol use among female sex workers and male clients: An integrative review of global literature. Alcohol Alcohol..

[12] Ao T.T., Sam N.E., Masenga E.J., Seage G.R., Kapiga S.H. (2006). Human immunodeficiency virus type 1 among bar and hotel workers in northern Tanzania: The role of alcohol, sexual behavior, and herpes simplex virus type 2. Sex. Transm. Dis..

[13] Chersich M.F., Bosire W., King’ola N., Temmerman M., Luchters S. (2014). Effects of hazardous and harmful alcohol use on HIV incidence and sexual behaviour: A cohort study of Kenyan female sex workers. Glob. Health.

[14] Chersich M.F., Luchters S.M., Malonza I.M., Mwarogo P., King’ola N., Temmerman M. (2007). Heavy episodic drinking among Kenyan female sex workers is associated with unsafe sex, sexual violence and sexually transmitted infections. Int. J. STD AIDS.

[15] Vandepitte J., Weiss H.A., Bukenya J., Nakubulwa S., Mayanja Y., Matovu G., Kyakuwa N., Hughes P., Hayes R., Grosskurth H. (2013). Alcohol use, mycoplasma genitalium, and other stis associated with HIV incidence among women at high risk in Kampala, Uganda. J. Acq. Immune Defic. Syndr..

[16] Schwitters A., Swaminathan M., Serwadda D., Muyonga M., Shiraishi R.W., Benech I., Mital S., Bosa R., Lubwama G., Hladik W. (2015). Prevalence of rape and client-initiated gender-based violence among female sex workers: Kampala, Uganda, 2012. AIDS Behav..

[17] L’Engle K.L., Mwarogo P., Kingola N., Sinkele W., Weiner D.H. (2014). A randomized controlled trial of a brief intervention to reduce alcohol use among female sex workers in Mombasa, Kenya. J. Acquir. Immune Defic. Syndr..

[18] Vandepitte J., Bukenya J., Weiss H.A., Nakubulwa S., Francis S.C., Hughes P., Hayes R., Grosskurth H. (2011). HIV and other sexually transmitted infections in a cohort of women involved in high-risk sexual behavior in Kampala, Uganda. Sex. Transm. Dis..

[19] Mbonye M., Rutakumwa R., Weiss H., Seeley J. (2014). Alcohol consumption and high risk sexual behaviour among female sex workers in Uganda. Afr. J. AIDS Res..

[20] Mbonye M., Nakamanya S., Nalukenge W., King R., Vandepitte J., Seeley J. (2013). “It is like a tomato stall where someone can pick what he likes”: Structure and practices of female sex work in Kampala, Uganda. BMC Public Health.

[21] Mayfield D., McLeod G., Hall P. (1974). The CAGE questionnaire: Validation of a new alcoholism screening instrument. Am. J. Psychiatry.

[22] Ewing J.A. (1984). Detecting alcoholism. The CAGE questionnaire. J. Am. Med. Assoc..

[23] Bohn M.J., Babor T.F., Kranzler H.R. (1995). The alcohol use disorders identification test (AUDIT): Validation of a screening instrument for use in medical settings. J. Stud. Alcohol.

[24] Localio A.R., Margolis D.J., Berlin J.A. (2007). Relative risks and confidence intervals were easily computed indirectly from multivariable logistic regression. J. Clin. Epidemiol..

[25] Hahn J.A., Fatch R., Wanyenze R.K., Baveewo S., Kamya M.R., Bangsberg D.R., Coates T.J. (2014). Decreases in self-reported alcohol consumption following HIV counseling and testing at Mulago hospital, Kampala, Uganda. BMC Infect. Dis..

[26] Maisto S.A., Clifford P.R., Davis C.M. (2007). Alcohol treatment research assessment exposure subject reactivity effects: Part II. Treatment engagement and involvement. J. Stud. Alcohol Drugs.

[27] Yadav G., Saskin R., Ngugi E., Kimani J., Keli F., Fonck K., Macdonald K.S., Bwayo J.J., Temmerman M., Moses S. (2005). Associations of sexual risk taking among Kenyan female sex workers after enrollment in an HIV-1 prevention trial. J. Acquir. Immune Defic. Syndr..

[28] Mongi A.S., Baisley K., Ao T.T., Chilongani J., Aguirre-Andreasen A., Francis S.C., Shao J., Hayes R., Kapiga S. (2013). Factors associated with problem drinking among women employed in food and recreational facilities in northern Tanzania. PLoS ONE.

[29] Ao T.T., Sam N., Kiwelu I., Mahal A., Subramanian S.V., Wyshak G., Kapiga S. (2011). Risk factors of alcohol problem drinking among female bar/hotel workers in Moshi, Tanzania: A multi-level analysis. AIDS Behav..

[30] Fisher J.C., Cook P.A., Sam N.E., Kapiga S.H. (2008). Patterns of alcohol use, problem drinking, and HIV infection among high-risk African women. Sex. Transm. Dis..

[31] Wechsberg W.M., Luseno W.K., Lam W.K. (2005). Violence against substance-abusing South African sex workers: Intersection with culture and HIV risk. AIDS Care.

[32] Peltzer K., Seoka P., Raphala S. (2004). Characteristics of female sex workers and their HIV/AIDS/STI knowledge, attitudes and behaviour in semi-urban areas in South Africa. Curationis.

[33] Zachariah R., Spielmann M.P., Harries A.D., Nkhoma W., Chantulo A., Arendt V. (2003). Sexually transmitted infections and sexual behaviour among commercial sex workers in a rural district of Malawi. Int. J. STD AIDS.

[34] Lavreys L., Chohan B., Ashley R., Richardson B.A., Corey L., Mandaliya K., Ndinya-Achola J.O., Kreiss J.K. (2003). Human herpesvirus 8: Seroprevalence and correlates in prostitutes in Mombasa, Kenya. J. Infect. Dis..

[35] Chege M.N., Kabiru E.W., Mbithi J.N., Bwayo J.J. (2002). Childcare practices of commercial sex workers. East Afr. Med. J..

[36] Wilson D., Chiroro P., Lavelle S., Mutero C. (1989). Sex worker, client sex behaviour and condom use in Harare, Zimbabwe. AIDS Care.

[37] Izulla P., McKinnon L.R., Munyao J., Karanja S., Koima W., Parmeres J., Kamuti S., Kioko R., Nagelkerke N., Gakii G. (2013). HIV postexposure prophylaxis in an urban population of female sex workers in Nairobi, Kenya. J. Acquir. Immune Defic. Syndr..

[38] Hahn J.A., Emenyonu N.I., Fatch R., Muyindike W.R., Kekiibina A., Carrico A.W., Woolf-King S., Shiboski S. (2016). Declining and rebounding unhealthy alcohol consumption during the first year of HIV care in rural Uganda, using phosphatidylethanol to augment self-report. Addiction.

[39] Asiimwe S.B., Fatch R., Emenyonu N.I., Muyindike W.R., Kekibiina A., Santos G.M., Greenfield T.K., Hahn J.A. (2015). Comparison of traditional and novel self-report measures to an alcohol biomarker for quantifying alcohol consumption among HIV-infected adults in sub-Saharan Africa. Alcohol. Clin. Exp. Res..

[40] Francis J.M., Weiss H.A., Helander A., Kapiga S.H., Changalucha J., Grosskurth H. (2015). Comparison of self-reported alcohol use with the alcohol biomarker phosphatidylethanol among young people in northern Tanzania. Drug Alcohol Depend..

[41] Pisa P.T., Vorster H.H., Kruger A., Margetts B., Loots du T. (2015). Association of alcohol consumption with specific biomarkers: A cross-sectional study in South Africa. J. Health Popul. Nutr..

[42] Bien T.H., Miller W.R., Tonigan J.S. (1993). Brief interventions for alcohol problems: A review. Addiction.

[43] Clifford P.R., Maisto S.A. (2000). Subject reactivity effects and alcohol treatment outcome research. J. Stud. Alcohol.

[44] Grodensky C.A., Golin C.E., Ochtera R.D., Turner B.J. (2012). Systematic review: Effect of alcohol intake on adherence to outpatient medication regimens for chronic diseases. J. Stud. Alcohol Drugs.

[45] Hahn J.A., Woolf-King S.E., Muyindike W. (2011). Adding fuel to the fire: Alcohol’s effect on the HIV epidemic in sub-Saharan Africa. Curr. HIV/AIDS Rep..

[46] Parry C.D. (2005). A review of policy-relevant strategies and interventions to address the burden of alcohol on individuals and society in South Africa. Afr. J. Psychiatry.

[47] Heather N. (2010). Breaking new ground in the study and practice of alcohol brief interventions. Drug Alcohol Rev..

[48] Burke B.L., Arkowitz H., Menchola M. (2003). The efficacy of motivational interviewing: A meta-analysis of controlled clinical trials. J. Consult. Clin. Psychol..

[49] Mathes T., Antoine S.L., Pieper D. (2014). Adherence-enhancing interventions for active antiretroviral therapy in sub-Saharan Africa: A systematic review and meta-analysis. Sex. Health.

[50] Morojele N.K., Kitleli N., Ngako K., Kekwaletswe C.T., Nkosi S., Fritz K., Parry C.D. (2014). Feasibility and acceptability of a bar-based sexual risk reduction intervention for bar patrons in Tshwane, South Africa. SAHARA J.

[51] Chersich M.F., Luchters S., Ntaganira I., Gerbase A., Lo Y.R., Scorgie F., Steen R. (2013). Priority interventions to reduce HIV transmission in sex work settings in sub-Saharan Africa and delivery of these services. J. Int. AIDS Soc..

[52] Wechsberg W.M., Luseno W.K., Lam W.K., Parry C.D., Morojele N.K. (2006). Substance use, sexual risk, and violence: HIV prevention intervention with sex workers in Pretoria. AIDS Behav..

